# Association Between the Serum Level of Asprosin and Metabolic Parameters in Adult Growth Hormone Deficiency: A Cross-Sectional Study

**DOI:** 10.1155/ije/9735508

**Published:** 2024-11-19

**Authors:** Hongbo Yang, Meiping Chen, Shanshan Liu, Yuelun Zhang, Linjie Wang, Lian Duan, Fengying Gong, Huijuan Zhu, Hui Pan

**Affiliations:** ^1^Key Laboratory of Endocrinology of National Health Commission, Department of Endocrinology, State Key Laboratory of Complex Severe and Rare Diseases, Peking Union Medical College Hospital, Chinese Academy of Medical Science and Peking Union Medical College, No. 1 Shuaifuyuan, Dongcheng, Beijing, China; ^2^Medical Research Center, Peking Union Medical College Hospital, Chinese Academy of Medical Sciences and Peking Union Medical College, No. 1 Shuaifuyuan, Dongcheng, Beijing, China

**Keywords:** adult growth hormone deficiency, asprosin, metabolic syndrome

## Abstract

**Objective:** Adult growth hormone deficiency (AGHD) is characterized by central adiposity and metabolic disorders. Asprosin, a newly discovered adipokine, plays a crucial role in connecting adipose tissue function with the development of metabolic syndrome. This study aims to evaluate the circulating levels of asprosin in AGHD patients and explore the potential correlation between asprosin levels and various metabolic parameters.

**Subjects and Methods:** Forty male patients with AGHD (mean age: 33.5 ± 9.5 yrs and mean BMI: 25.0 ± 4.5 kg/m^2^) and forty age-, gender-, and BMI-matched non-AGHD controls were enrolled. Medical history, anthropometric parameters (weight, height, waist circumference), and biochemical and hormonal investigations were collected from the electronic medical record system. Fat mass, fat percentage, and fat-free mass (FFM) were evaluated by bioelectrical impedance. Serum levels of asprosin were measured by ELISA.

**Results:** Patients with AGHD demonstrated notably increased waist-to-hip ratios, triglyceride levels, and decreased HDL-cholesterol levels compared with the control group. In additionally, AGHD patients exhibited significantly higher serum levels of asprosin compared with controls (*p*=0.039). A notable association was observed between serum asprosin levels and FFM, triglycerides, and HDL-cholesterol levels in the whole population.

**Conclusions:** Our study highlights distinct metabolic alterations in AGHD patients when matched for BMI with controls and investigates variations in serum asprosin levels for the first time. These findings have significant implications for identifying potential biomarkers for metabolic syndrome risk in AGHD patients and informing future treatment approaches.

## 1. Introduction

Adult growth hormone deficiency (AGHD) is associated with various metabolic disorders [1]. Studies have shown that patients with AGHD frequently have abnormalities in their glucose metabolism, including decreased glucose tolerance and increased insulin resistance [2, 3]. In addition, dyslipidemia and central obesity have been associated with AGHD, which makes for a poor lipid profile and worsens insulin resistance [4]. The incidence of nonalcoholic fatty liver disease is also reported to be much higher than that of the general population [5]. The risk of cardiovascular diseases may arise due to these metabolic changes in AGHD [6–8]. Recombinant human growth hormone treatment lowers the risk of cardiovascular risks by reducing fat deposition, increasing lean body mass, restoring lipid profiles, and lowering systemic inflammation [9]. The primary impact of growth hormone (GH) on lipid metabolism is the stimulation of lipolysis, leading to an increase in the release of free fatty acids (FFAs) and glycerol from adipose tissue into circulation [10]. Circulating insulin-like growth factor-1 (IGF-1) is crucial in insulin sensitivity in peripheral tissues [11]. Numerous studies have demonstrated the involvement of GH/IGF-1 in regulating the body's energy balance through potential mechanisms such as modulating oxidative stress and inflammatory responses, influencing muscle mass and function, adipose tissue development, and central regulation of appetite [12, 13]. Therefore, GH/IGF-1 deficiency is the primary causative factor for metabolic disorders in AGHD. However, the intricate relationship between GH/IGF-1 and metabolic disorders necessitates further investigation to comprehensively elucidate the underlying mechanisms linking these hormones to the development of metabolic syndrome.

Adipokines, bioactive molecules secreted by adipose tissue, have gained recognition as crucial contributors to the pathophysiology of metabolic syndrome [14]. These adipocyte-derived factors exert diverse effects on various organs and tissues, influencing metabolic homeostasis, inflammation, endothelial function, and insulin sensitivity [15]. Recent studies have indicated a potential association between adipokines and metabolic syndrome in patients with AGHD [16, 17]. Investigating alterations in the expression of these adipokines in AGHD patients with metabolic syndrome will provide valuable insights and novel targets for therapeutic interventions to improve long-term prognosis.

Asprosin, a novel adipokine secreted in response to fasting, exerts multifaceted effects on metabolic tissues [18]. By interacting with hepatocytes through an identified olfactory G-protein coupled receptor and potentially other unknown receptors, asprosin stimulates glucose secretion [19]. Furthermore, it influences appetite by targeting agouti-related peptide-expression neurons in the central nervous system [20]. In skeletal muscle, asprosin-induced endoplasmic reticulum stress and inflammation compromise insulin signaling, leading to increased insulin resistance [21]. Recent studies have reported elevated circulating levels of asprosin in individuals with obesity and type 2 diabetes, demonstrating robust association with relevant clinical markers [22]. However, studies investigating the specific impact of asprosin on patients with AGHD are currently lacking.

In this cross-sectional study, serum asprosin levels were measured in middle-aged patients with AGHD to investigate the impact of GH/IGF-1 deficiency on asprosin levels and to examine potential biomarkers of metabolic disorders associated with GH/IGF-1 deficiency by establishing correlations between serum asprosin levels and metabolic parameters. The results indicate that dysregulation of GH and IGF-1 may disrupt serum asprosin levels, potentially contributing to the development of metabolic disorders. Our results suggest a potential mechanism between GH/IGF-1 deficiency and metabolic syndrome development and identify a potential therapeutic target. These findings hold significant clinical implications for guiding future treatment strategies for AGHD patients with metabolic complications.

## 2. Subjects and Methods

### 2.1. Subjects

In this single-center study, forty consecutive male AGHD patients were enrolled in Peking Union Medical College Hospital from December 2018 to December 2021. The diagnosis of AGHD was based on established clinical criteria from the American Endocrine Society [23]. For suspected GHD patients, an insulin tolerance test (ITT) is recommended to confirm the diagnosis of GHD. GHD can be diagnosed when IGF-1 levels are lower than normal for age and sex, in conjunction with evident characteristic of GHD and deficiencies in the other three pituitary hormone axes. Among the patients studied, two patients had a peak value of growth hormone less than 5 ng/mL in the ITT. The other 38 patients did not undergo ITT since the level of IGF-1 was below the lower limit of the normal range and three or more pituitary axis were deficient. Patients with multiple pituitary hormone deficiencies received replacement therapy with prednisone, thyroxine, or testosterone as clinically indicated.

Forty non-AGHD male controls matched with age and BMI were enrolled during the same period. The inclusion criteria for controls were as follows: (1) the absence of any pituitary or hypothalamic disease and normal functioning of the anterior pituitary and (2) no prior history of diabetes mellitus, hypertension, or hyperlipidemia, as well as the absence of any current medication usage. The study was conducted in compliance with the principles of the Declaration of Helsinki of the World Medical Congress. The study protocol was approved by the Ethics Committees of Peking Union Medical College Hospital (K3454).

### 2.2. Clinical and Laboratory Data Collection

To evaluate metabolic risk factors in AGHD patients, we chose measures related to obesity, such as BMI, waist circumference indicating visceral obesity, and body composition to reflect obese body composition. In addition, routine blood biochemistry, glucose metabolism, and lipid levels were assessed. Evaluation indices for insulin sensitivity, including the Homeostasis Model Assessment of Insulin Resistance (HOMA-IR), a widely utilized measure for insulin resistance globally [24], and the triglyceride–glucose (TyG) index, were also included. The TyG index serves as a novel indicator for predicting metabolic disorders and cardiovascular disease [25]. The clinical data, including medical history, age, height, weight, waist circumference (WC), hip circumference (HC), and body composition data from Bioimpedance, were all retrieved from electronic medical records. The laboratory tests of all subjects were measured by collecting venous blood after an 8–12-h overnight fasting. Liver function, kidney function, lipid profile (total cholesterol [TC], low and high-density lipoprotein cholesterol [LDL-c and HDL-c], triglyceride [TG]), fasting blood glucose (FBG), fasting insulin (FINS), HbA1c, testosterone (T), and thyroid function were assessed using standardized methods at the clinical laboratory department of PUMCH. HOMA-IR was calculated as HOMA-IR = [(fasting blood sugar (mmol/L)) × (fasting plasma insulin (pmol/L))]/22.5. TyG index was calculated as TyG index = Ln [fasting TGs (mg/dL) × fasting blood sugar (mg/dL)/2]. IGF-1 was determined by automatic chemiluminescence enzyme immunoassay (IMMULITE 2000, Siemens Healthcare Diagnostics). The IGF-1 SD score (SDS) was calculated according to previously published data from healthy Chinese adults [26]. For those receiving T replacement, serum T concentration was measured before the next injection.

### 2.3. Measurement of Circulating Asprosin Levels

Serum levels of asprosin were measured by using an ELISA kit (Wuhan Youersheng Trading Co., Ltd, Cat. SEA332Hu), following the protocols provided by the manufacturer. The detection range of human asprosin is 0.156–10 ng/mL. The calculated intrabatch coefficients of variation are < 10% and inter-batch variation is < 12%. In brief, the antibody to asprosin was encapsulated in microwells. Standards or serum samples were added to the microwells, respectively, in which the asprosin binds to the antibody attached to the solid-phase carrier. Then, the biotinylated asprosin antibody was added, the unbound biotinylated antibody was washed, and then horseradish peroxidase-labeled affinity protein was added. The tetramethylbenzidine (TMB) substrate was added to develop the color after washing thoroughly again. TMB was converted into a blue color under the catalysis of peroxidase and converted into a final yellow color under the action of acid. The absorbance (O.D. value) was measured at 450 nm using an enzyme meter, and the sample concentration was calculated.

### 2.4. Statistical Analysis

This study used SPSS 27.0 for data statistical analysis. The categorical variable is expressed in percentage, and the measurement data is expressed in the mean ± standard deviation or median (quartile). The normality test is carried out using the Shapiro–Wilk test and the P-P chart for all measurement data. The independent sample *t*-test and nonparametric Mann–Whitney *U* test compare the AGHD and control groups for measurement data according to the data distribution. Spearman bivariate correlation analysis was used to investigate the correlation between the serum asprosin level and clinical parameters. A *p* value less than 0.05 is considered statistically significant.

## 3. Results

### 3.1. Demographic and Clinical Characteristics of the Study Groups

Table 1 presents the basic disease characteristics of 40 male AGHD patients. These patients exhibited a median disease duration of 19.8 (4.8, 26.2). Regarding the etiology, the cohort consisted of 18 cases of pituitary dysplasia (45.0%), ten cases of craniopharyngiomas (25.0%), eight cases of nonfunctional pituitary adenomas (20.0%), three cases of intracranial germinal cell tumors (7.5%), and one case of pituitary abscess (2.5%). Upon evaluation of anterior pituitary function, adrenal insufficiency was observed in 29 (72.5%) patients, central hypothyroidism in 36 patients, and hypogonadotropic hypogonadism in 29 patients (72.5%). In addition, 14 patients (35.0%) had diabetes insipidus. Among the cohort, one patient was treated with antiglycemic drugs, and three patients received lipid-lowering drugs.

Table 2 shows the anthropometric parameters, body composition, and metabolic and hormonal characteristics of the two studied groups. As anticipated, AGHD patients showed significant reductions in height, IGF-1, IGF-1SDS, HDL-c, and elevated levels of TGs compared with the control group. Despite being matched with age and BMI, the waist-to-hip ratios was significantly higher in the AGHD group compared with the control group (0.91 ± 0.06 vs. 0.84 ± 0.05, *p*=0.011). The levels of T and FT4 in the AGHD group were lower in the AGHD group but remained within the normal ranges. In addition, the level of creatinine (Cr) was significantly lower in the AGHD group compared with the control group. However, there were no significant differences between the two groups in terms of body composition indices such as FAT%, FAT mass, FFM, TBW, or other metabolic indices including FBG, FINS, HbA1c, HOMA-IR, LDL-c, TC, TyG index, and uric acid (UA).

### 3.2. Increased Serum Asprosin Levels in AGHD Patients and the Correlation to Metabolic Parameters

**Figure 1:**
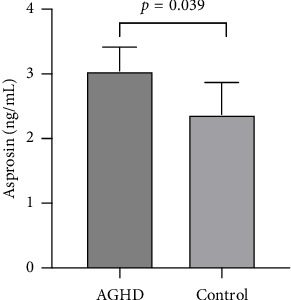
Serum asprosin levels in AGHD versus healthy subjects.

**Figure 2:**
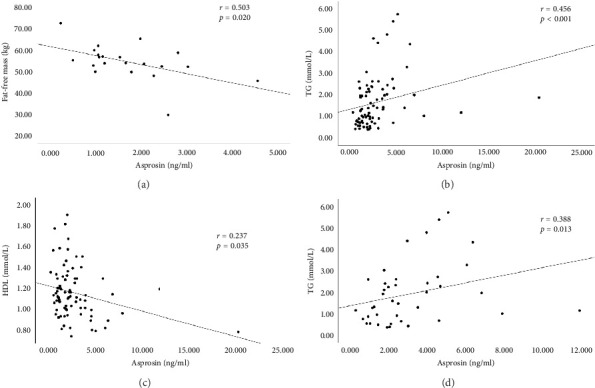
(a–c) Correlations between serum asprosin levels and fat free mass, triglycerides or HDL-cholesterol in all the populations evaluated (AGHD and healthy controls) and (d) correlations between serum asprosin levels and triglycerides in AGHD.

Serum asprosin levels (ng/mL) were significantly higher in AGHD patients than in controls ((2.83 ± 2.33 (1.47, 4.36) vs. 1.82 (1.06, 2.72), *p*=0.039; Figure 1). No significant differences were described between asprosin and age, BMI, blood pressure, glutamic pyruvic transaminase (ALT), Cr, and IGF-1, respectively (Table 3). The association between asprosin and metabolic parameters in AGHD and the overall analyzed population was further analyzed. We found that serum asprosin levels were significantly negatively correlated with FFM, TBW, and HDL-c in the whole population (Figure 2). Moreover, a strong positive correlation between serum asprosin and TG levels was noted in both AGHD (Spearman R coefficient = 0.388, *p*=0.013) and the whole population (Spearman R coefficient = 0.456, *p* < 0.001; Figure 2).

## 4. Discussion

In this pilot study, we aimed to investigate the serum asprosin levels in AGHD patients for the first time. In addition, we explored the potential correlation between serum asprosin levels and metabolic parameters in AGHD patients. Our findings revealed several important observations. First, AGHD patients exhibited significantly higher waist-to-hip ratios and notable alterations in the metabolic status compared with BMI-matched controls. Second, our data demonstrated a significant elevation in serum asprosin levels among AGHD patients. Furthermore, we identified significant associations between serum asprosin levels and key metabolic parameters such as FFM, TGs, and HDL-c levels. Overall, our data provide valuable insights into the metabolic changes associated with AGHD and propose asprosin as a promising biomarker for identifying AGHD patients at risk of developing metabolic syndrome.

It is widely recognized that AGHD patients commonly exhibit clinical signs of being overweight, altered metabolic profiles, and a high prevalence of metabolic syndrome [27]. However, the precise differentiation between the “metabolic syndrome of AGHD” and the conventional metabolic syndrome has yet to be fully elucidated. Our results suggest that significant differences in waist-to-hip ratios and lipid levels were observed between AGHD and non-AGHD overweight controls with the same BMI. Therefore, it is necessary to search for the characteristic metabolic biomarkers of AGHD patients. Previous studies have also shown that although metabolic syndrome and AGHD have similar clinical and biochemical parameters, there are differences in low-grade inflammation types between the two syndromes [2, 28, 29]. Adipokines have emerged as a pivotal player in the pathophysiology of metabolic syndrome, making them a focal point of research in recent years. This growing interest reflects the potential to delve into the alterations in expression and functions of these adipokines in metabolic syndrome [14]. Research has demonstrated that the GH/IGF-1 axis has an impact on adipokine secretion, which in turn mediates metabolic effects of the GH/IGF-1 axis [30, 31]. This suggests a potential link between impaired adipokine function and the development of metabolic abnormalities in AGHD patients. Multiple studies have examined the role of leptin and adiponectin in GHD, with varying results. Some studies have indicated elevated leptin levels [32, 33] and decreased response to growth hormone replacement therapy (GHRT) [33], as well as lower adiponectin levels compared with patients with acromegaly but similar to those of healthy individuals [34]. Furthermore, recent research has shown that adipsin levels are elevated in patients with AGHD compared with controls and are significantly associated with cardiometabolic risk factors, which might be a good marker for the occurrence and development of cardiovascular disease in AGHD patients [16]. However, no data currently exist regarding the influence of asprosin levels on metabolic parameters specifically in AGHD patients.

This study found that serum asprosin levels were significantly higher in AGHD patients than non-AGHD controls. Asprosin, as a diabetogenic adipokine, is mainly derived from white adipose tissue and is associated with glucose metabolism, lipid distribution, IR, and *β* cell function [18]. AGHD patients experience a long-term growth hormone deficit, leading to diminished fat breakdown and increased fat accumulation. Within our cohort, although the BMI indicated only overweight levels, AGHD patients exhibited a notable increase in waist-to-hip ratios. This finding suggests visceral fat accumulation occurs early in adulthood among AGHD patients. In addition, these patients demonstrated alterations in their lipid profiles, characterized by elevated TG levels and reduced HDL-c levels. Considering these metabolic statuses, it is plausible that the observed increase in asprosin levels among AGHD patients is closely associated with these metabolic changes. Moreover, our study revealed a positive correlation between serum asprosin levels and TGs in individuals with AGHD and the general population and a negative correlation with FFM and HDL-c in the AGHD group. Consistent with previous research of 131 patients with metabolic syndrome and 162 age-matched healthy controls, serum asprosin levels were positively associated with TGs and negatively associated with HDL-c. They were independently and positively associated with TGs in multiple linear regression analysis [35]. In addition, a positive correlation between asprosin and TGs was also found in patients with type 2 diabetes [36, 37]. These findings suggest that asprosin levels play are influential in regulating lipid metabolism in AGHD patients and may serve as a valuable biomarker for AGHD patients at risk for metabolic syndrome. Our research group previously investigated the role of asprosin in patients with acromegaly [38]. In contrast to GHD, acromegaly is characterized by a chronic excess of growth hormone. Interestingly, a decrease in serum asprosin levels has been associated with prolonged elevation of growth hormone levels. Based on this observation, the increased serum asprosin levels observed in AGHD patients may be attributed to the long-term effects of low growth hormone levels. The mechanism by which asprosin affects abnormal lipid metabolism in AGHD patients has yet to be fully elucidated. It has been reported that asprosin has important effects on regulating appetite, glucose metabolism, IR, apoptosis, and so on through the central nervous system, peripheral tissues, and organs [39]. Centrally, asprosin was able to cross the blood-brain barrier and directly acted on hunger-stimulating neurons known as agouti-related peptide neurons (AgRP) by a cAMP-dependent pathway in the hypothalamic arcuate nucleus (ARH), activating the feeding neural circuit to stimulate appetite which might lead to excessive energy absorption and obesity [40]. Peripherally, asprosin induces hepatic glucose production and directly affects insulin signaling, leading to insulin resistance, inflammation, and endoplasmic reticulum stress, leading to lipid metabolism disorders and the onset of obesity [21, 39, 41]. However, metabolic changes' intricate influence on asprosin in AGHD patients requires further investigation. Additional research is needed to unravel the complexities of this relationship.

Numerous studies have shown a direct correlation between asprosin and blood glucose and insulin resistance in people with obesity, type 2 diabetes, and metabolic syndrome [42]. The negative regulation of asprosin secretion by blood glucose has been found in both mouse and human studies that the serum asprosin level decreases with the increase of blood glucose, and asprosin can also induce the rapid decomposition and release of liver glycogen to increase blood glucose [18, 43]. A study in nondiabetic obese people has found that asprosin concentrations are positively correlated with multiple insulin resistance indices such as the TyG index [42]. Our study found no significant correlation between asprosin and FBG, FINS, or insulin resistance indicators such as HOMA-IR and TyG index. Furthermore, no correlation was observed between asprosin levels and BMI in the overweight population, including AGHD patients and the overall study population. The inconsistent findings regarding the correlation between asprosin and glucose metabolism indices in AGHD patients can be explained by a disease associated with low GH levels. It is known that GH has an anti-insulin effect, and a short-term reduction in GH levels can diminish this insulin antagonism and enhance insulin sensitivity [10]. Besides, the AGHD patients included in our study were relatively young and did not exhibit significant glucose metabolism disorders. Considering the interplay between GH and insulin, this mutual regulation could explain the lack of correlation between asprosin and glucose metabolism indexes in AGHD patients. In addition, it is well known that BMI is a rough indicator for assessing the ratio of weight to height and cannot objectively reflect the body fat composition [44], just as the differences in lipid indexes between AGHD patients and BMI-matched controls described in our data, which may also be one of the reasons for the lack of correlation between serum asprosin and BMI in AGHD patients. It must be mentioned that the inclusion of the population to match BMI also brings a particular selection bias.

Several limitations should be acknowledged in this study. First, the cross-sectional study design restricts the ability to establish causality and only permits the assessment of associations. Future longitudinal studies are needed to elucidate the temporal relationship between asprosin levels and metabolic changes in individuals with AGHD. In addition, the constraints of sample size and potential confounding factors may impede the generalizability of the findings. Enhancing the findings' reliability and applicability could be achieved by implementing a large-scale, high-quality cohort study involving multiple centers. It is important to acknowledge that efforts were made to carefully match confounding factors, including age, gender, and BMI, between the study and control groups in our research. Thus, despite these constraints, our data provide a robust basis for future investigations.

In conclusion, our research findings indicated that AGHD patients exhibit elevated waist-to-hip ratios and altered metabolic status compared with BMI-matched controls. Serum levels of asprosin may serve as a valuable biomarker for identifying AGHD patients at risk of developing metabolic syndrome.

## Figures and Tables

**Table 1:** Clinical characteristics of AGHD patients.

	AGHD (*n* = 40)
Duration of disease (yr)	19.8 (4.8, 26.2)

*Etiology of AGHD*	
Pituitary hypoplasia	18 (45.0%)
Craniopharyngioma	10 (25.0%)
Nonfunctioning pituitary adenoma	8 (20.0%)
Intracranial germinoma	3 (7.5%)
Pituitary abscess	1 (2.5%)

*Deficiency of pituitary function*	
Corticotropic	29 (72.5%)
Thyrotropic	36 (90.0%)
Gonadotropic	29 (72.5%)
Diabetes insipidus	14 (35.0%)

*Concomitant medication*	
Antidiabetic	1 (2.5%)
Lipid lowering	3 (7.5%)

**Table 2:** Anthropometric, biochemical, and hormonal characteristics of the study populations.

	Healthy controls	AGHD baseline	*p*_0_ value
Height (cm)	176.5 ± 5.3	172.0 (168.0, 175.0)	< 0.001
Weight (kg)	76.6 ± 8.8	73.5 (60.5, 82.3)	0.123
Waist (cm)/hip (cm) ratio	0.84 ± 0.05	0.91 ± 0.06	0.011
FAT (%)	22.57 ± 4.19	25.12 ± 5.53	0.143
FAT mass (kg)∗	16.63 ± 4.44	17.78 ± 6.20	0.574
FFM (kg)∗	55.96 ± 4.47	50.44 ± 15.4	0.471
TBW (kg)∗	40.98 ± 3.28	36.92 ± 11.26	0.198
ALT (U/L)	21.5 (17.0, 33.3)	26.5 (14.5, 37.0)	0.310
Cr (U/L)	82.3 ± 10.2	70.0 (64.0, 80.8)	< 0.001
UA (*μ*mol/L)	399.7 ± 66.7	401.0 ± 91.7	0.943
GLU (mmol/L)	5.18 ± 0.44	5.02 ± 0.45	0.115
FINS (*μ*U/mL)	7.60 (6.68, 11.33)	10.87 ± 6.35	0.661
HbA1c (%)	5.3 (5.1, 5.5)	5.3 (5.0, 5.8)	0.540
TC (mmol/L)	4.92 ± 0.81	4.91 ± 1.20	0.964
TG (mmol/L)	1.05 (0.74, 1.88)	1.55 (0.81, 2.57)	0.073
HDL-c (mmol/L)	1.24 ± 0.24	1.08 (0.95, 1.17)	0.001
LDL-c (mmol/L)	3.02 ± 0.66	2.94 ± 0.92	0.664
HOMA-IR	1.62 (1.38, 2.68)	2.02 (1.34, 3.12)	0.767
TyG index	4.57 ± 0.30	4.69 ± 0.38	0.145
IGF-1 (ng/mL)	220.5 (184.5, 253.0)	64.0 (45.3, 94.8)	< 0.001
IGF-1 SDS	0.37 ± 0.66	−3.05 ± 1.70	< 0.001
IGF-1/INS	27.62 ± 9.91	7.16 (4.43, 11.39)	< 0.001
T (ng/mL)	4.46 (3.44, 5.20)	2.61 (0.16, 4.16)	< 0.001
FT4 (ng/dL)	1.30 ± 0.16	1.02 ± 0.35	< 0.001

Abbreviations: ALT, glutamic pyruvic transaminase; Cr, creatinine; FAT%, fat mass percentage; FBG, fasting blood glucose; FFM, fat-free mass; FINS, fasting insulin; HbA1c, glycated hemoglobin A1c; HDL-c, high-density lipoprotein cholesterol; HOMA-IR, homeostasis model assessment of insulin resistance; IGF-1, insulin-like growth factor 1; LDL-c, low-density lipoprotein cholesterol; SDS, SD score; T, testosterone; TBW, total body water; TC, total cholesterol; TG, triglyceride; TyG index, triglyceride–glucose index; UA, uric acid; WHR, waist-to-hip ratio.

^∗^Missing data. Data were calculated from 5 AGHD patients and 16 controls.

**Table 3:** Bivariate correlation analysis of serum asprosin levels.

Variables	*r*	*p* value
Age	0.216	0.055
BMI	−0.06	0.595
SBP	−0.052	0.656
DBP	0.003	0.981
Waist (cm)/hip (cm) ratio	0.162	0.449
Fat (%)	0.329	0.057
Fat mass	0.091	0.647
FFM (kg)	−0.503⁣^∗^	0.020
TBW (kg)	−0.503⁣^∗^	0.020
ALT	0.094	0.406
Cr	−0.117	0.302
Glu (mmol/L)	0.052	0.649
FINS (*μ*U/mL)	−0.03	0.845
HbA1c (%)	0.007	0.958
TyG	0.194	0.087
HOMA-IR	0.132	0.389
TC (mmol/L)	0.163	0.147
TG (mmol/L)	0.456⁣^∗∗^	< 0.001
LDL (mmol/L)	0.012	0.916
HDL (mmol/L)	−0.237⁣^∗^	0.035
IGF1	−0.209	0.063
IGF1SDS	−0.044	0.701
IGF-1/INS	−0.321	0.030

⁣^∗^*p* < 0.05.

⁣^∗∗^*p* < 0.01.

## Data Availability

The data that support the findings of this study are available from the corresponding author upon reasonable request.

## References

[1] Melmed S. (2019). Pathogenesis and Diagnosis of Growth Hormone Deficiency in Adults. *New England Journal of Medicine*.

[2] Basile U., Bruno C., Napodano C. (2018). Plasmatic Free Light Chains as Inflammatory Marker in Insulin Resistance: Comparison of Metabolic Syndrome with Adult Growth Hormone Deficiency. *BioFactors*.

[3] Luger A., Mattsson A. F., Koltowska-Häggström M. (2012). Incidence of Diabetes Mellitus and Evolution of Glucose Parameters in Growth Hormone-Deficient Subjects During Growth Hormone Replacement Therapy: a Long-Term Observational Study. *Diabetes Care*.

[4] Møller N., Jørgensen J. O. L. (2009). Effects of Growth Hormone on Glucose, Lipid, and Protein Metabolism in Human Subjects. *Endocrine Reviews*.

[5] Doycheva I., Erickson D., Watt K. D. (2022). Growth Hormone Deficiency and NAFLD: An Overlooked and Underrecognized Link. *Hepatol Commun*.

[6] Gazzaruso C., Gola M., Karamouzis I., Giubbini R., Giustina A. (2014). Cardiovascular Risk in Adult Patients With Growth Hormone (GH) Deficiency and Following Substitution With GH--an Update. *Journal of Clinical Endocrinology and Metabolism*.

[7] Giovannini L., Tirabassi G., Muscogiuri G., Di Somma C., Colao A., Balercia G. (2015). Impact of Adult Growth Hormone Deficiency on Metabolic Profile and Cardiovascular Risk [Review]. *Endocrine Journal*.

[8] Di Somma C., Scarano E., Savastano S., Savanelli M. C., Pivonello R., Colao A. (2017). Cardiovascular Alterations in Adult GH Deficiency. *Best Practice & Research Clinical Endocrinology & Metabolism*.

[9] Maison P., Griffin S., Nicoue-Beglah M. (2004). Impact of Growth Hormone (GH) Treatment on Cardiovascular Risk Factors in GH-Deficient Adults: A Metaanalysis of Blinded, Randomized, Placebo-Controlled Trials. *Journal of Clinical Endocrinology and Metabolism*.

[10] Huang Z., Huang L., Waters M. J., Chen C. (2020). Insulin and Growth Hormone Balance: Implications for Obesity. *Trends in Endocrinology and Metabolism*.

[11] Friedrich N., Thuesen B., Jørgensen T. (2012). The Association between IGF-I and Insulin Resistance: a General Population Study in Danish Adults. *Diabetes Care*.

[12] Al-Samerria S., Radovick S. (2023). Exploring the Therapeutic Potential of Targeting GH and IGF-1 in the Management of Obesity: Insights from the Interplay between These Hormones and Metabolism. *International Journal of Molecular Sciences*.

[13] Al-Samerria S., Radovick S. (2021). The Role of Insulin-like Growth Factor-1 (IGF-1) in the Control of Neuroendocrine Regulation of Growth. *Cells*.

[14] Unamuno X., Gómez-Ambrosi J., Rodríguez A., Becerril S., Frühbeck G., Catalán V. (2018). Adipokine Dysregulation and Adipose Tissue Inflammation in Human Obesity. *European Journal of Clinical Investigation*.

[15] Mechanick J. I., Zhao S., Garvey W. T. (2016). The Adipokine-Cardiovascular-Lifestyle Network: Translation to Clinical Practice. *Journal of the American College of Cardiology*.

[16] Wang Y., Zheng X., Xie X., Qian W., Zhang L., Ren W. (2019). Correlation of Increased Serum Adipsin With Increased Cardiovascular Risks in Adult Patients with Growth Hormone Deficiency. *Endocrine Practice*.

[17] Vergani E., Bruno C., Gavotti C., Oliva A., Currò D., Mancini A. (2022). Increased Levels of Plasma Neudesin in Adult Growth Hormone Deficiency and Their Relationship with Plasma Liver-Expressed Antimicrobial Peptide-2 Levels: a Cross-Sectional Study. *Journal of Endocrinological Investigation*.

[18] Romere C., Duerrschmid C., Bournat J. (2016). Asprosin, a Fasting-Induced Glucogenic Protein Hormone. *Cell*.

[19] Li E., Shan H., Chen L. (2019). OLFR734 Mediates Glucose Metabolism as a Receptor of Asprosin. *Cell Metabolism*.

[20] Feng B., Liu H., Mishra I. (2023). Asprosin Promotes Feeding through SK Channel-Dependent Activation of AgRP Neurons. *Science Advances*.

[21] Jung T. W., Kim H.-C., Kim Ho U. (2019). Asprosin Attenuates Insulin Signaling Pathway Through PKC*δ*-Activated ER Stress and Inflammation in Skeletal Muscle. *Journal of Cellular Physiology*.

[22] Ugur K., Erman F., Turkoglu S. (2022). Asprosin, Visfatin and Subfatin as New Biomarkers of Obesity and Metabolic Syndrome. *European Review for Medical and Pharmacological Sciences*.

[23] Molitch M. E., Clemmons D. R., Malozowski S., Merriam G. R., Vance M. L., Society E. (2011). Evaluation and Treatment of Adult Growth Hormone Deficiency: An Endocrine Society Clinical Practice Guideline. *Journal of Clinical Endocrinology and Metabolism*.

[24] Matthews D. R., Hosker J. P., Rudenski A. S., Naylor B. A., Treacher D. F., Turner R. C. (1985). Homeostasis Model Assessment: Insulin Resistance and Beta-Cell Function From Fasting Plasma Glucose and Insulin Concentrations in Man. *Diabetologia*.

[25] Mohd Nor N. S., Lee SoJ., Bacha F., Tfayli H., Arslanian S. (2016). Triglyceride Glucose Index as a Surrogate Measure of Insulin Sensitivity in Obese Adolescents with Normoglycemia, Prediabetes, and Type 2 Diabetes Mellitus: Comparison with the Hyperinsulinemic-Euglycemic Clamp. *Pediatric Diabetes*.

[26] Zhu H., Xu Y., Gong F. (2017). Reference Ranges for Serum Insulin-like Growth Factor I (IGF-I) in Healthy Chinese Adults. *PLoS One*.

[27] Molitch M. E., Clemmons D. R., Malozowski S. (2006). Evaluation and Treatment of Adult Growth Hormone Deficiency: An Endocrine Society Clinical Practice Guideline. *Journal of Clinical Endocrinology and Metabolism*.

[28] Mancini A., Di Segni C., Bruno C. (2018). Oxidative Stress in Adult Growth Hormone Deficiency: Different Plasma Antioxidant Patterns in Comparison With Metabolic Syndrome. *Endocrine*.

[29] Currò D., Vergani E., Bruno C., Comi S., D’Abate C., Mancini A. (2020). Plasmatic Lipocalin-2 Levels in Chronic Low-Grade Inflammation Syndromes: Comparison Between Metabolic Syndrome, Total and Partial Adult Growth Hormone Deficiency. *BioFactors*.

[30] Witkowska-Sędek E., Rumińska M., Stelmaszczyk-Emmel A., Majcher A., Pyrżak B. (2018). The Associations between the Growth Hormone/Insulin-Like Growth Factor-1 Axis, Adiponectin, Resistin and Metabolic Profile in Children With Growth Hormone Deficiency Before and During Growth Hormone Treatment. *Acta Biochimica Polonica*.

[31] Orrù S., Nigro E., Mandola A. (2017). A Functional Interplay Between IGF-1 and Adiponectin. *International Journal of Molecular Sciences*.

[32] Joaquin C., Aguilera E., Granada M. L. (2008). Effects of GH Treatment in GH-Deficient Adults on Adiponectin, Leptin and Pregnancy-Associated Plasma Protein-A. *European Journal of Endocrinology*.

[33] Fisker S., Vahl N., Hansen T. B. (1997). Serum Leptin Is Increased in Growth Hormone—Deficient Adults: Relation to Body Composition and Effects of Placebo-Controlled Growth Hormone Therapy for 1 Year. *Metabolism*.

[34] Fukuda I., Hizuka N., Ishikawa Y. (2004). Serum Adiponectin Levels in Adult Growth Hormone Deficiency and Acromegaly. *Growth Hormone & IGF Research*.

[35] Hong T., Li J.-Y., Wang Y.-D. (2021). High Serum Asprosin Levels Are Associated with Presence of Metabolic Syndrome. *International Journal of Endocrinology*.

[36] Zhong M., Tian X., Sun Q. (2023). Correlation of Asprosin and Nrg-4 with Type 2 Diabetes Mellitus Complicated with Coronary Heart Disease and the Diagnostic Value. *BMC Endocrine Disorders*.

[37] Zhang L., Chen C., Zhou N., Fu Y., Cheng X. (2019). Circulating Asprosin Concentrations Are Increased in Type 2 Diabetes Mellitus and Independently Associated with Fasting Glucose and Triglyceride. *Clinica Chimica Acta*.

[38] Ke X., Duan L., Gong F. (2020). Serum Levels of Asprosin, a Novel Adipokine, Are Significantly Lowered in Patients With Acromegaly. *International Journal of Endocrinology*.

[39] Farrag M., Ait Eldjoudi D., González-Rodríguez M. (2022). Asprosin in Health and Disease, a New Glucose Sensor with Central and Peripheral Metabolic Effects. *Frontiers in Endocrinology*.

[40] Duerrschmid C., He Y., Wang C. (2017). Asprosin Is a Centrally Acting Orexigenic Hormone. *Nature Medicine*.

[41] Zou J., Xu C., Zhao Z.-W., Yin S.-H., Wang G. (2022). Asprosin Inhibits Macrophage Lipid Accumulation and Reduces Atherosclerotic Burden by Up-Regulating ABCA1 and ABCG1 Expression via the p38/Elk-1 Pathway. *Journal of Translational Medicine*.

[42] Mirr M., Braszak-Cymerman A., Ludziejewska A. (2023). Serum Asprosin Correlates With Indirect Insulin Resistance Indices. *Biomedicines*.

[43] Wiecek M., Szymura J., Maciejczyk M., Kantorowicz M., Szygula Z. (2018). -A Comparison between Sexes. *Frontiers in Physiology*.

[44] Götherström G., Svensson J., Koranyi J. (2001). A Prospective Study of 5 Years of GH Replacement Therapy in GH-Deficient Adults: Sustained Effects on Body Composition, Bone Mass, and Metabolic Indices. *Journal of Clinical Endocrinology and Metabolism*.

